# Short Sleep Duration in the First Years of Life and Obesity/Overweight at Age 4 Years: A Birth Cohort Study

**DOI:** 10.1016/j.jpeds.2015.09.074

**Published:** 2016-01

**Authors:** Camila S.E. Halal, Alicia Matijasevich, Laura D. Howe, Iná S. Santos, Fernando C. Barros, Magda L. Nunes

**Affiliations:** 1 PhD Program of Medicine and Health Sciences, Pontificia Universidade Católica do Rio Grande do Sul, Porto Alegre, Brazil; 2 Conceição Hospital Group, Porto Alegre, Brazil; 3 Department of Preventive Medicine, School of Medicine, University of São Paulo, São Paulo, Brazil; 4 Postgraduate Epidemiology Program, Federal University of Pelotas, Pelotas, Brazil; 5 Medical Research Council Integrative Epidemiology Unit at the University of Bristol, School of Social and Community Medicine, University of Bristol, Bristol, United Kingdom; 6 Postgraduate Program of Health and Behavior, Catholic University of Pelotas, Pelotas, Brazil; 7 Division of Neurology, Pontificia Universidade Católica do Rio Grande do Sul School of Medicine and Brain Institute (INSCER), Porto Alegre, Brazil

**Keywords:** BMI, Body mass index, PR, Prevalence ratio

## Abstract

**Objective:**

To investigate whether short sleep duration from the first year of life influenced weight at an early age.

**Study design:**

During 2004, children born in Pelotas, Brazil, were enrolled in a cohort study. Sleeping habits during the previous 2 weeks were assessed, and the children were weighed and measured at 1-, 2-, and 4-year follow-ups. Overweight and obesity at 4 years were defined according to World Health Organization z-scores for body mass index for age. Short sleep duration was defined as fewer than 10 hours of sleep per night at any follow-up.

**Results:**

Out of the 4263 live births, 4231 were recruited. The prevalence of short sleep duration at any follow-up from 1-4 years of age was 10.1%. At 4 years of age, 201 children were obese (5.3%), and 302 (8%) were overweight. Among short sleepers, the prevalence ratio for overweight/obesity after adjusting for maternal and children's characteristics was 1.32 (1.03; 1.70).

**Conclusions:**

Children who slept for fewer than 10 hours per night at any follow-up from 1-4 years of age were more likely to be overweight or obese at 4 years of age, despite their sociodemographic and sleep characteristics.

Experimental studies conducted mainly among adults have suggested that curtailment of sleep may be linked to weight gain, possibly through elevation of cortisol and ghrelin levels, along with reduction of leptin levels, thereby leading to increased hunger and reduced energy expenditure.^1^, ^2^, ^3^, ^4^ Although individual variability in sleep need is considered to be a major determinant of the number of hours slept per day, the amount of sleep considered to be physiological is around 12-15 hours for infants and 11-14 hours for toddlers.^5^ Regarding nighttime sleep, the average amount expected is approximately 12 hours at 1 year of age and 11 hours at 4 years of age.^6^ Studies have indicated that children currently have at least 30 fewer minutes of sleep per day than the recommended amount.^7^ This seems to be due to later onset times for sleeping, while the time schedule for waking up in the morning is maintained.^6^ This finding has been observed from an early age and may also play an important role in weight gain.^8^, ^9^

There is divergent evidence regarding the relationship between short sleep duration and weight status in childhood,^10^, ^11^, ^12^, ^13^, ^14^ and a lack of evidence linking very early exposure to short sleep duration and weight changes before school age.^2^, ^15^ The present study aimed to investigate the association between short sleep duration at any time between 1 and 4 years of age and overweight or obesity at 4 years of age.

## Methods

This study was developed in Pelotas, a city in southern Brazil with a population of around 328 000 inhabitants,^16^ of whom approximately 95% live in the urban area. Over 99% of deliveries take place in 1 of the 5 city hospitals.

The 2004 Pelotas Birth Cohort, conducted through the Postgraduate Epidemiology Program at the Federal University of Pelotas, was designed to collect information from all children born from January 1, 2004, to December 31, 2004, to mothers living in the urban area of the municipality. More information on the study design and results can be found elsewhere.^17^, ^18^, ^19^ Briefly, mothers were recruited in the hospital within 24 hours of delivery by trained fieldworkers and were interviewed using a structured questionnaire that asked for information on demographic, socioeconomic, and behavioral characteristics, along with reproductive history, antenatal care, and illnesses.

Children were evaluated at birth and subsequently at follow-up home visits at 1, 2, and 4 years of age. Data on growth, development, morbidity, and feeding habits were collected, as well as social and demographic information.

The information collected at the perinatal interview included maternal age in complete years (<20, 20-29, 30-39, ≥40); skin color (based on interviewer observation, and classified as white/black/other); mother's schooling level in complete years of formal education (0, 1-4, 5-7, 8-10, ≥11); living with a partner; number of antenatal care appointments (1-4, 5-8, ≥9); parity (1, ≥2); mother's body mass index (BMI) at the beginning of pregnancy with weight from prenatal records or reported by the mother; and height measured by the interviewer (<18.5, 18.5-24.9, 25-29.9, ≥30 kg/m^2^). Smoking, alcohol consumption, high blood pressure, and diabetes during gestation were reported by the mother. Socioeconomic status was categorized according to the Brazilian criteria of economic classification, which take into account household assets and the education level of the family head and are divided in 5 groups (A-E), such that A is the wealthiest.

Type of delivery was obtained from hospital records. Gestational age (<37, ≥37 weeks) was estimated according to the last menstrual period if it was consistent with birth weight, length, and head circumference (based on curves for gestational age)^20^; if inconsistent or unknown, newborn maturity was estimated using the Dubowitz method.^21^

Newborns were measured in length by the interviewers using an infantometer with 1 mm precision. Birth weight in g (≥4000, 2501-3999, 1500-2500, <1500) was collected from hospital records, which had been derived from electronic pediatric scales with 10 g precision that had been checked weekly by the study staff using standard weights. Breastfeeding practices were assessed at 1 year of age.

Data on sleep characteristics were collected at 1, 2, and 4 years of age, regarding the child's usual bedtime and wake up time during the 2 weeks preceding the interview. Total nighttime sleep duration was based on the parents' responses to questioning on what time the child was put to bed, how long it took the child to fall asleep (sleep latency), and what time the child usually woke up the following morning. Because sleep latency was on average 20 minutes at every follow-up, long sleep latency time was operationally defined by the authors as more than 50% longer than the mean (>30 minutes). Children who slept for fewer than 10 hours per night were considered to be short sleepers.^22^ Because only 9 children (0.25%) slept less than 10 hours at all visits, the variable “short sleep duration between 12 and 48 months” was built such that it included children who slept for fewer than 10 hours in at least 1 of the follow-ups.

The parents were asked by whom the child was put to bed (mother, father, mother and father, other, nobody) and about the presence of night waking, number of nights with awakenings (0, 1, ≥2/wk), number of awakenings per night (0, 1, ≥2), use of a pacifier throughout the night, room or bed sharing, and number (0, 1, ≥2) and duration (<1, ≥1 hour) of daytime naps. The respondents were asked to judge the quality of their child's sleep (excellent, very good, good, fair, poor). Information on nightmares or night terrors was sought at the 2- and 4-year follow-ups, and on television viewing at nighttime at the 4-year visit (<1, ≥1 hour).

At the 1-year visit, children were weighed on their mother's lap using an electronic scale with 100 g precision, after their mother had been weighed while wearing light clothing. At the 2 and 4-year follow-ups, they were weighed on the same scale, standing on their own. Length was assessed in the recumbent position at 1 and 2 years, by means of an infantometer with 1 mm precision. At the 4-year visit, height was measured using a portable stadiometer with 1 mm precision, which was developed for this study.

BMI was calculated, and BMI z-scores determined. Children were classified as overweight or obese in accordance with World Health Organization z-score BMI-for-age metrics.^23^ Children with a BMI z-score between 2 and 2.99 SDs above the World Health Organization cut-point were considered overweight, and those with a z-score of 3 SDs or more were considered obese.

The variables explored as being potential confounders of the association between short sleep duration and overweight/obesity were the antenatal maternal characteristics, those of the child at birth, and at 1 year of age. Other sleep variables measured at age 1 year (start of the exposure period) like night waking, sleep latency, sleep quality, and daytime naps were explored as potential confounders and mediators (Figure; available at www.jpeds.com).

This decision was based on a conceptual framework describing the postulated hierarchical relationships between exposures.^24^ To be included in the model, variables had to be associated with fewer than 10 hours of sleep per night between the ages of 1 and 4 years and with overweight, obesity, or both at 4 years of age, with a *P* value of <.20, but without being in the causal pathway between shorter sleep duration and overweight/obesity.^25^

For multivariable analyses, Poisson regression with robust variance was used. The results were expressed as prevalence ratios (PRs) and their respective 95% CIs. Three analysis models were used: crude analysis (model 1); model 1 + mother's skin color and education level (model 2); and model 2 + sleep latency, number of night waking, and duration of day naps at 1 year of age (model 3).

In addition to using “any sleep duration between ages 1 and 4” as our main exposure, we also wished to assess the time-varying nature of the associations as a way of checking for reverse causality. We, therefore, assessed the association of: (1) short sleep duration at 1 year of age and overweight/obesity at 2 years of age, with adjustment for overweight/obesity at 1 year of age, mother's skin color, and schooling; and (2) short sleep duration at 2 years of age and overweight/obesity at 4 years of age, with adjustment for overweight/obesity at 1 and 2 years of age, short sleep duration at 1 year of age, mother's skin color, and schooling.

All of the analyses were made using the Stata statistical software, v 12.1 (StataCorp LP, College Station, Texas).

The Medical Research Ethics Committee of the Federal University of Pelotas, which is affiliated to the Brazilian National Committee for Research Ethics, approved the study protocols of all the waves of the cohort. The mothers signed a written informed consent statement.

## Results

Among the mothers of the 4263 live births, 4231 agreed to take part in the study. At 1, 2, and 4 years of age, the numbers of children followed up were 3907 (94%), 3869 (93.5%), and 3799 (92%), respectively.

Maternal and newborn characteristics among children with and without short sleep duration can be found on Tables I and II (Table II available at www.jpeds.com). The overall prevalence of overweight or obesity at 4 years of age was 13.3% (n = 503), that of overweight alone was 8% (n = 302), and obesity was 5.3% (n = 201). The prevalence of short sleep duration was 4.1% (n = 159), 3.5% (n = 136), and 4.1% (n = 156) at 1, 2, and 4 years of age, respectively (Table III). Short sleep duration at any time between the ages of 1 and 4 years showed prevalence of 10.1% (n = 367).

The mean durations of sleeping at 1, 2, and 4 years of age were 812.9 ± 113.1, 800.5 ± 111.6, and 797.4 ± 133.0 minutes, respectively. Among short sleepers, the mean durations of sleeping at 1, 2, and 4 years were of age 447.2 ± 133.1, 405.7 ± 191.3, and 411.7 ± 193.1 minutes, respectively. Table III shows the participants' sleep characteristics.

The prevalence of short sleep duration was higher among the children of mothers with black skin color and with fewer years of education, and among those who used a pacifier throughout the night, were put to bed by both the mother and the father, took longer to fall asleep, did not nap during the day or napped for shorter periods, and whose mothers considered that they had worse quality of sleep at 1 year of age (Table IV; available at www.jpeds.com).

The prevalence of overweight/obesity at 48 months of age was higher among children in families presenting higher socioeconomic conditions, those whose mothers were white, more highly educated, those whose mothers were overweight or obese at the beginning of pregnancy, those who were firstborns, and those born at gestational ages of more than 37 weeks. Cesarean delivery and higher birth weight also correlated positively with overweight or obesity at 4 years of age. Regarding sleep characteristics, using a pacifier throughout the night, being put to bed by both the mother and the father, and bed sharing at the age of 1 year were also correlated with higher prevalence of overweight/obesity.

The crude PR for overweight/obesity at 4 years of age among children with short sleep duration at any time between the ages of 1 and 4 years, in comparison with those without short sleep duration, was 1.26 (95% CI 0.98-1.62; Table V). After full adjustment for confounders, the PR was 1.32 (95% CI 1.03-1.70).

Our sensitivity analysis in evaluation of time-varying associations provided evidence that our findings were not due to reverse causality. After controlling for mother's skin color and schooling and overweight/obesity at 1 year, short sleep duration at 1 year had a PR of 1.14 (95% CI 0.68-1.91; *P* = .620) for overweight/obesity at 2 years. After allowing for mother's skin color and schooling, sleep duration at age 1 year, and overweight/obesity at ages 1 and 2 years, short sleep duration at 2 years of age had a PR of 1.90 (95% CI 1.28-2.84; *P* = .01) for overweight/obesity at age 4 years.

To check whether sleep duration during the 24-hour period was also associated with obesity at the age of 4 years, analyses considering total sleep duration (nighttime and daytime sleep) as the exposure were run. Prevalence of short sleep duration during the 24-hour period in at least one of the follow-ups was 7.0% (n = 196). After adjusting for maternal skin color and education, who put the child to bed, and number of daytime naps, children who slept less than 10 hours in 24 hours had a PR of 1.44 (95% CI 1.03-2.02; *P* = .032), close to that observed when only nighttime sleep duration was analyzed.

## Discussion

In the population studied, sleeping for fewer than 10 hours per night at any time between the ages of 1 and 4 years was associated with a 32% higher risk of being overweight or obese by the age of 4 years, in comparison with those without short sleep duration. The highest risk in relation to short sleep duration was found at the age of 2 years. This finding is important because it suggests that children's short sleep duration may influence weight status and, thus, contribute toward exposing the child to overweight and its consequences from a very early stage in life.

The observed prevalence of overweight or obesity (13.3%) was higher in this population than in Brazilian national estimates, and it was also higher than the average for the south of the country, where Pelotas is located.^26^ In the US, longitudinal data on infant weight status show similar prevalence: 13.9% between the ages of 2 and 5 years in 2003-2004,^27^ and 12.1% in that same age group in 2009-2010.^28^ Between previous cohort studies in Pelotas, there was a clearly increasing trend in children's weight status; the prevalence of overweight at 1 year of age was 6.5% in the 1982 cohort, and this rose to 9.4% 11 years later, in the 1993 birth cohort.^29^ Similar prevalence also has been observed in other Latin American countries, such as Mexico and Bolivia, and also in developing countries in the Middle East and Africa.^30^

With regard to the observed association between short sleep duration and overweight/obesity, other studies, conducted in China, Germany, and the US have reported similar findings.^2^, ^15^, ^31^

Our study has several strengths and limitations. One of the limitations is the lack of information on metabolic variables. Studies assessing the metabolic mechanism through which short sleep duration may influence weight gain have mainly been directed toward the adult population. In addition, except duration of exclusive breastfeeding, feeding habits present in infancy such as age of introduction and type of complementary feeding that could independently influence weight status, were not explored as potential confounders.

Although objective sleep-evaluation methods such as actigraphy or polysomnography were not used, information on what time the children were put to bed and how long it took them to fall asleep was taken into consideration when calculating total sleep time, thus, diminishing the probability of sleep time overestimation. In addition, there is evidence that among preschoolers, mothers' reports are consistent with actigraphy measurements for total sleep time.^32^

The strengths of this study include its population-based design with a high number of children included at birth and very low rates of losses during follow-up visits, and our analysis strategy that provided evidence that the associations were not due to reverse causality. Questions regarding the child's usual behavior in terms of bedtime and wake-up time over the 2 weeks preceding the interview diminished the chances that answers might be influenced by acute illnesses, which may interfere with the child's bedtime routine and sleep quality, or by alterations to the routine relating to catch-up sleep on weekends or holidays.

Further research, using objective sleep-evaluation methods and laboratory evaluations, is needed. Interventions using sleep hygiene and sleep education strategies may be useful for analyzing the effect that increasing total sleep time may have on preventing and managing overweight or obesity among preschool children.^33^

## Figures and Tables

**Figure:**
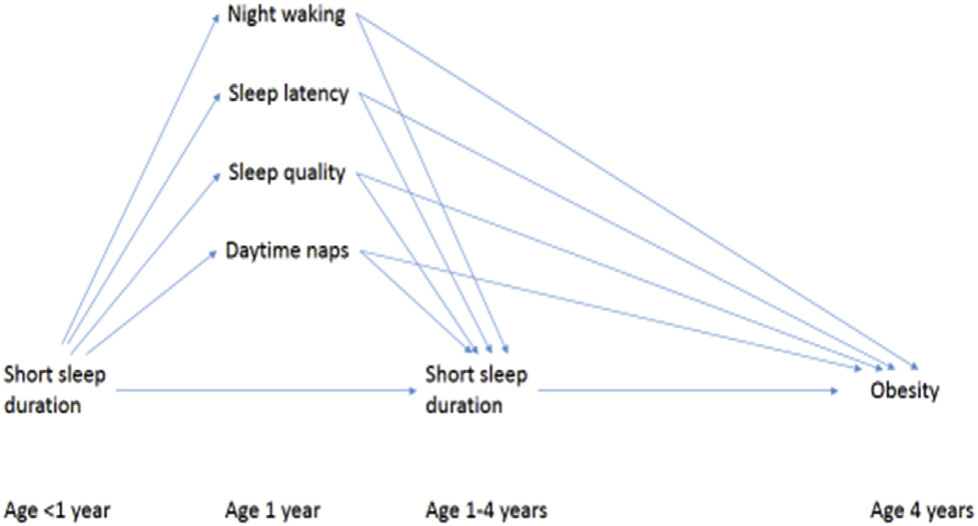
Analytical model to evaluate the association between early exposure to short sleep duration and overweight/obesity at age 4 years. More nights with awakenings, longer sleep latency, worst sleep quality, and daytime naps could, throughout the time, function as mediators to that association.

**Table II:** Type of delivery, birth weight, and demographic characteristics at birth in the whole cohort, and among the children with and without short sleep duration

Characteristics	N (%)	Children without short sleep duration	Children with short sleep duration
Type of delivery			
Vaginal	2309 (54.6)	1788 (54.6)	196 (53.4)
Cesarean	1922 (45.4)	1490 (45.4)	171 (46.6)
Sex			
Male	2196 (51.9)	1687 (51.5)	208 (56.7)
Female	2035 (48.1)	1591 (48.5)	159 (43.3)
Birth weight (g)			
≥4000	201 (4.7)	154 (4.7)	21 (5.7)
2501-3999	3599 (85.1)	2825 (86.2)	312 (85.0)
1500-2500	369 (8.7)	274 (8.4)	31 (8.5)
≤1500	62 (1.5)	25 (0.7)	3 (0.8)
Gestational age (wk)			
<37	613 (14.5)	448 (13.7)	53 (14.4)
≥37	3618 (85.5)	2830 (86.3)	314 (85.6)

**Table IV:** Prevalence of short sleep duration at any time between 1 and 4 years of age and of overweight or obesity at 4 years of age according to maternal and child characteristics

Variables	Prevalence of short sleep duration at any time between 1 and 4 y of age (%)	Prevalence of overweight or obesity at 4 y of age (%)
Economic status	*P* = .645	*P* < .001
A	8.6	12.1
B	8.0	15.1
C	10.4	17.0
D	9.7	8.8
E	8.7	4.5
Maternal age (y)	*P* = .716	*P* = .364
<20	10.9	10.8
20-29	10.1	12.5
30-39	9.2	13.1
≥40	9.5	15.5
Mother's skin color	*P* = .031	*P* = .160
White	9.3	13.1
Black	12.6	10.8
Other	9.7	10.5
Mother's schooling (y)	*P* = .037	*P* < .001
0	15.2	3.0
1-4	8.4	8.6
5-7	12.5	9.0
8-10	9.1	15.7
≥11	9.3	17.3
Parity	*P* = .979	*P* < .001
1	10.0	15.8
≥2	10.0	10.3
BMI start of gestation (kg/m^2^)	*P* = .934	*P* < .001
<18.5	11.7	5.2
18.5-24.9	9.8	10.4
25-29.9	10.1	15.9
≥30	10.7	27.3
Gestational age at birth (wk)	*P* = .680	*P* = .004
<37	10.5	8.5
≥37	9.9	13.1
Type of delivery	*P* = .655	*P* < .001
Vaginal	9.8	10.0
Cesarean	10.2	15.4
Sex	*P* = .067	*P* = .351
Male	10.9	12.9
Female	9.0	11.9
Birth weight (g)	*P* = .986	*P* = .001
≥4000	10.7	19.5
2501-3999	9.9	12.6
1500-2500	10.2	6.6
≤1500	10.7	10.7
Breastfed at 1 y of age	*P* = .400	*P* = .298
No	10.4	13.7
Yes	9.5	12.5
Use of pacifier throughout night at 1 y of age	*P* = .007	*P* = .012
No	9.4	13.9
Yes	12.8	10.4
Who puts the child to bed at 1 y of age	*P* = .031	*P* = .049
Mother	9.3	13.4
Father	12.8	12.8
Mother and father	17.0	21.7
Other	11.3	11.8
Nobody	11.6	9.2
Sleeps alone in the bedroom at 1 y of age	*P* = .150	*P* = .342
No	10.3	13.2
Yes	7.2	15.4
Bed sharing at 1 y of age	*P* = .220	*P* < .001
No	9.5	15.5
Yes	10.7	10.7
Sleep latency at 1 y of age (min)	*P* = .003	*P* = .201
≤30	9.5	13.1
>30	14.4	15.4
Woke up during the night at 1 y of age	*P* = .446	*P* = .247
No	10.6	12.4
Yes	9.8	13.8
Number of nights with awakenings at 1 y of age	*P* = .231	*P* = .264
0	10.6	12.4
1	6.7	16.4
≥2	10.1	13.5
Number of awakenings per night at 1 y of age	*P* = .511	*P* = .079
0	10.6	12.4
1	9.2	15.3
≥2	10.3	12.6
Number of daytime naps at 1 y of age	*P* = .042	*P* = .156
0	21.6	2.7
1	9.4	13.6
≥2	10.3	13.3
Duration of daytime naps at 1 y of age (h)	*P* = .016	*P* = .251
<1	12.1	12.3
≥1	9.3	13.8
Sleep quality at 1 y of age (maternal perception)	*P* = .001	*P* = .731
Excellent	8.8	13.0
Very good	8.0	15.3
Good	9.7	13.1
Fair	11.5	12.7
Poor	19.3	12.7

**Table I:** Maternal antenatal demographic, socioeconomic, reproductive, and behavioral characteristics in the whole cohort and among the mothers of children with and without short sleep duration

Characteristics	N (%)	Children without short sleep duration	Children with short sleep duration
Skin color			
White	3090 (73.0)	2415 (73.7)	249 (67.9)
Black	846 (20.0)	638 (19.4)	93 (25.3)
Other	295 (7.0)	225 (6.9)	25 (6.8)
Age (y)			
<20	800 (18.9)	599 (18.3)	73 (19.9)
20-29	2108 (49.8)	1620 (49.4)	184 (50.2)
30-39	1184 (28.0)	952 (29.0)	97 (26.4)
≥40	139 (3.3)	107 (3.3)	13 (3.5)
Schooling (full y)			
0 y	43 (1.0)	28 (0.9)	5 (1.4)
1-4	612 (14.6)	469 (14.4)	43 (11.9)
5-7	1136 (27.1)	845 (26.0)	121 (33.4)
8-10	1050 (25.1)	849 (26.2)	85 (23.5)
≥11	1347 (32.2)	1056 (32.5)	108 (29.8)
Lives with husband/partner (yes)	3536 (83.6)	2759 (84.2)	311 (84.7)
Economic status∗			
A	65 (2.0)	54 (2.0)	5 (1.8)
B	515 (15.8)	418 (15.7)	37 (13.0)
C	1128 (34.6)	934 (35.1)	108 (38.2)
D	1230 (37.6)	995 (37.3)	108 (38.2)
E	327 (10.0)	264 (9.9)	25 (8.8)
Parity			
1	1666 (39.4)	1293 (39.4)	144 (39.2)
≥2	2565 (60.6)	1984 (60.6)	223 (60.8)
Number of antenatal care appointments			
1-4	418 (9.9)	293 (8.9)	34 (9.2)
5-8	1761 (41.6)	1397 (42.6)	154 (42.0)
≥9	2052 (48.5)	1588 (48.5)	179 (48.8)
BMI at beginning of pregnancy (kg/m^2^)			
<18.5	86 (3.6)	68 (3.7)	9 (4.4)
18.5-24.9	1421 (59.8)	1110 (59.9)	120 (58.2)
25-29.9	653 (27.5)	508 (27.4)	57 (27.7)
≥30	215 (9.1)	167 (9.0)	20 (9.7)
High blood pressure during pregnancy (yes)	1010 (23.9)	775 (23.7)	96 (26.2)
Diabetes during pregnancy (yes)	126 (3.0)	99 (3.0)	12 (3.3)
Smoking during pregnancy (yes)	1162 (27.5)	860 (26.2)	120 (32.7)
Alcohol consumption during pregnancy (yes)	140 (3.3)	108 (3.3)	9 (2.5)

∗ Brazilian criteria of economic classification.

**Table III:** Sleep behavior of children at 1, 2, and 4 years of age, over the 2 weeks preceding the interview

Sleep characteristics	1 y (%)	2 y (%)	4 y (%)
Sleep deprivation (yes)	159 (4.1)	136 (3.5)	156 (4.1)
Use of pacifier throughout night (yes)	755 (19.3)	865 (22.4)	466 (12.3)
Who usually puts the child to bed			
Mother	3032 (77.6)	2567 (66.4)	1757 (46.3)
Father	317 (8.1)	333 (8.6)	248 (6.5)
Mother and Father	115 (3.0)	186 (4.8)	158 (4.2)
Other	263 (6.7)	245 (6.3)	233 (6.1)
Nobody	179 (4.6)	537 (13.9)	1403 (36.9)
Sleeps alone in the room (no)	3671 (94.0)	3622 (93.6)	3299 (86.8)
Shares the bed (yes)	1793 (45.9)	1868 (48.3)	1722 (45.3)
Sleep latency			
≤30 min	3490 (89.5)	3506 (90.8)	3428 (90.7)
>30 min	408 (10.5)	352 (9.1)	351 (9.3)
Woke up during the night (yes)	2516 (64.4)	1917 (49.6)	1776 (46.6)
Number of nights with awakenings			
0	1390 (35.6)	1953 (50.5)	2025 (53.4)
1	227 (5.8)	333 (8.6)	346 (9.1)
≥2	2286 (58.6)	1579 (40.9)	1422 (37.5)
Number of awakenings per night			
0	1390 (35.7)	1951 (50.5)	2022 (53.2)
1	1157 (29.7)	1199 (31.0)	1369 (36.1)
≥2	1350 (34.6)	717 (18.5)	407 (10.7)
Number of naps during the d			
0	40 (1.0)	359 (9.3)	2265 (53.5)
1	1449 (37.1)	3380 (87.4)	1486 (35.2)
≥2	2414 (61.9)	127 (3.3)	480 (11.3)
Duration of naps			
<1 h	1001 (25.9)	388 (11.1)	169 (11.1)
≥1 h	2860 (74.1)	3113 (88.9)	1355 (88.9)
Child's sleep quality (mother's perception)			
Excellent	584 (15.0)	643 (16.6)	885 (23.3)
Very good	538 (13.7)	807 (20.9)	694 (18.3)
Good	1930 (49.4)	1818 (47.0)	1749 (46.0)
Fair	695 (17.8)	516 (13.3)	412 (10.8)
Poor	159 (4.1)	84 (2.2)	59 (1.6)
Nightmares/night terrors (yes)	-	522 (13.5)	527 (13.9)
Agitated sleep (yes)	-	1366 (35.3)	1326 (34.9)
Duration of watching television at night			
<1 h	-	-	1273 (35.4)
≥1 h	-	-	2320 (64.6)

**Table V:** Crude and adjusted PRs with 95% CIs for overweight or obesity at 4 years of age among children with short sleep duration at any time between 1 and 4 years of age

Models	PR (95% CI)	*P*
Model 1 = crude	1.26 (0.98; 1.62)	.061
Model 2 = model 1 + mother's skin color and schooling	1.32 (1.03; 1.70)	.024
Model 3 = model 2 + sleep characteristics measured at 1 y of age (sleep latency, number of night awakenings, and duration of daytime naps)	1.32 (1.03; 1.70)	.028
